# Sex differences in microvascular and Alzheimer’s disease‐related pathology in the medial temporal lobe: a quantitative neuropathological study

**DOI:** 10.1002/alz.088832

**Published:** 2025-01-03

**Authors:** Francesco Bax, Marteen L. van den Berg, Jan Oltmer, Corinne A. Auger, Alexandra N. Melloni, Theresa Connors, Alberto Serrano‐Pozo, Matt P. Frosch, Steven M. Greenberg, Bradley T. T Hyman, Susanne J. van Veluw, Valentina Perosa

**Affiliations:** ^1^ J. Philip Kistler Stroke Research Center, Massachusetts General Hospital, Harvard Medical School, Boston, MA USA; ^2^ MassGeneral Institute for Neurodegenerative Disease, Massachusetts General Hospital, Harvard Medical School, Charlestown, MA USA; ^3^ Department of Digital Health & Innovation, Vivantes Netzwerk für Gesundheit GmbH, Berlin, Germany Germany; ^4^ Neuropathology Service, C.S. Kubik Laboratory for Neuropathology, Massachusetts General Hospital, Harvard Medical School, Boston, MA USA; ^5^ Massachusetts General Hospital, Boston, MA USA

## Abstract

**Background:**

Alzheimer’s disease (AD) related pathologies (i.e., neurofibrillary tangles [NFTs], amyloid‐β plaques, and phosphorylated‐TAR‐DNA‐binding‐protein‐43 [pTDP‐43]) differ across sexes. However, the interaction between sex and cerebral small vessel disease (CSVD) (i.e., cerebral amyloid angiopathy [CAA] and arteriolosclerosis) on AD‐related pathologies has been less well characterized. The medial temporal lobe (MTL) is a crucial region in AD pathophysiology which harbors AD‐pathologies at an early disease stage and is vascularized by small vessels prone to CSVD. We therefore aimed to analyze the relationship between sex and CSVD on AD‐related pathologies in the MTL of a pathological AD cohort.

**Methods:**

The study included autopsy cases from the Massachusetts AD Research Center. One hemisphere was formalin‐fixed, samples from pre‐defined regions (hippocampal body and entorhinal cortex) were stained for luxol fast blue with hematoxylin&eosin, amyloid‐b, At8, and pTDP‐43. Deep‐learning models (Aiforiaâ) were used to obtain quantitative measures for burden of CAA, NFTs, and pTDP‐43 inclusions. Arteriolosclerosis (grade 0‐3) was determined in vessels >20mm Ø. Mixed effect models explored age and sex (fixed terms) across regions of interest (ROIs: hippocampus, parahippocampal gyrus, entorhinal cortex, amygdala) of the MTL on CSVD subtypes. Furthermore, interactions between sex and CSVD subtypes on AD‐related pathologies were tested. Finally, ApoE effect was evaluated in a subgroup with available genotyping.

**Results:**

157 autopsy cases (80.8±12.6y, 91 females) were included, ApoE status was available in 66/157. Females had higher arteriolosclerosis severity (odds ratio for grade 2/3 = 1.56, 95% confidence interval [CI] 1.11; 2.20, p = 0.01) and lower CAA burden (b = ‐0.08, 95%CI ‐0.12; ‐0.04, p<0.001), when adjusting for age and ROIs. Moreover, females had lower burden of amyloid‐b plaques (b = ‐0.25, 95%CI ‐0.46; ‐0.04, p = 0.02) and higher density of NFTs (b = 5.16, 95%CI 1.90; 8.41, p = 0.002). Inclusion of ApoE‐status confirmed these findings with higher effect‐size, and ApoE4 genotype interacted with female sex predicting higher NFT density when adjusting for arteriolosclerosis and CAA respectively (b = 16.2, 95%CI 4.2; 28.2, p = 0.008 and b = 16.3, 95%CI 3.37; 29.22, p = 0.01). No interaction effect was found between sex and CSVD on AD‐pathology.

**Conclusions:**

In this cohort, sex differentially affected microvascular and AD‐related pathologies in the MTL. ApoE4 genotype might act as an effect modifier of sex.

